# Part 1: profiling extra cellular matrix core proteome of human fetal nucleus pulposus in search for regenerative targets

**DOI:** 10.1038/s41598-020-72859-x

**Published:** 2020-09-24

**Authors:** Shanmuganathan Rajasekaran, Chitraa Tangavel, Niek Djuric, Muthurajan Raveendran, Dilip Chand Raja Soundararajan, Sharon Miracle Nayagam, Monica Steffi Matchado, K. S. Sri Vijay Anand

**Affiliations:** 1 Department of Spine Surgery, Ganga Hospital, 313, Mettuppalayam Road, Coimbatore, 641043 India; 2 Ganga Research Centre, No 91, Mettuppalayam Road, Coimbatore, 641030 India; 3 Department of Neurosurgery, Leiden University Medical Center, Leiden, The Netherlands; 4 Department of Plant Biotechnology, Tamil Nadu Agricultural University, Coimbatore, 641003 India

**Keywords:** Medical research, Preclinical research, Molecular biology, Proteomics

## Abstract

Intervertebral disc degeneration is accompanied by a loss of Extra-cellular matrix (ECM) due to an imbalance in anabolic and catabolic pathways. Identifying ECM proteins with anabolic and/or regenerative potential could be the key to developing regenerative therapies. Since human fetal discs grow and develop rapidly, studying these discs may provide valuable insights on proteins with regenerative potential. This study compares core matrisome of 9 fetal and 7 healthy adult (age 22–79) nucleus pulposus (NP), using a proteomic and bioinformatic approach. Of the 33 upregulated proteins in fetus NP’s, 20 of which were involved in ECM assembly pathways: fibromodulin, biglycan, heparan sulfate proteoglycan 2, chondroitin sulfate proteoglycan 4, procollagen C-endopeptidase enhancer and Collagen—type 1a1, 1a2, 6a1, 6a3, 11a1, 11a2, 12a1, 14a1 and 15a1. Moreover, 10 of the upregulated proteins were involved in growth pathways ‘PI3L-Akt signaling’ and ‘regulation of insulin like growth factor transport and uptake.’ Thrombospondin 1,3 and 4, tenascin C, matrilin-3, and collagen- type 1a1, 1a2, 6a1, 6a3 and 9a1. Additionally, matrillin-2 and ‘Collagen triple helix repeat containing 1’ were identified as possible regenerative proteins due to their involvement in ‘Regeneration’ and ‘tissue development’ respectively. In conclusion, the consistency of human fetal NP’s differs greatly from that of healthy adults. In view of these outcomes, the core matrisome of human fetal discs contains an abundant number of proteins that could potentially show regenerative properties, and their potential should be explored in future machinal experiments.

## Introduction

A major supportive structure of the human adult spine is the intervertebral disc (IVD). The IVD consists of three major components: firstly, the nucleus pulposus (NP), which is a gel-like structure located at the inner part of the IVD; secondly, the annulus fibrosus (AF), a fibrous structure which is layered around the NP, and at last, the cartilage endplate (CEP), which is a thin cartilage sheet between the disc and the vertebral body^1^. Together, these three structures create an elastic, hydrodynamic, weight bearing cushion for the spine, thereby allowing it to absorb compressive loads while remaining flexible^2–4^.

During aging, the structure and components of the IVD change, especially in the NP, since it has a great variety in ECM proteins^5,6^. The dry weight of a NP consists approximately 70% of ECM, in which collagen type 2 and proteoglycans are the two most abundant proteins types^6^. These proteoglycans integrate with the collagen fibrils and elastin to create a well-structured ECM that contributes to the strength and elasticity of the disc^4,7^. Besides, proteoglycans are highly charged and they are also responsible for the hydraulic pressure of the disc^4^. This pressure, in turn, is essential for its weight bearing capacity^5^. During aging, the disc tends to slowly lose its strength and elasticity. While in many cases, this does not cause serious problems; some discs will start to degenerate^4^. During disc degeneration (DD), catabolic pathways are upregulated and the anabolic pathways are downregulated^8^. This imbalance leads to a decrease in ECM protein content, which results in a reduction in size and strength and is often accompanied by inflammation^8,9^. Due to these changes, patients with DD usually suffer from low back pain, which is currently one of the leading causes of physical disability worldwide^10^. As of today, the only effective treatment for DD is surgery which urges the need for non-surgical-regenerative therapies. Such therapies could lead to major improvements in terms of both disability and healthcare costs.

As mentioned, the pathophysiology of this highly disabling disease involves a dysregulation of the catabolic/anabolic balance^8^. Therefore, it is crucial to explore all possible anabolic mechanisms that can be exploited. Recent research has focused on exploring these pathways in both healthy and degenerated discs^4,6,8^. Stem cells, proteins and genetic therapeutic options are being explored for regenerative potential in disc degeneration using animal models and in-vitro culture methods. However, their successfully integration in to human clinical practice is challenged by the intricate biology and homeostatic mechanisms of nucleus pulposus. Recently, nucleus pulposus has been identified to have two types of cells: notochordal and chondrocyte type cells. Notochordal cells play an important role in synthesizing proteoglycans and other ECM molecules^11^. They originate in the embryo, continue through fetal life, and have been found to substantially decrease during ageing. The age of disappearance differs greatly from species to species^12^, in humans this is believed to occur around age 10^13^. Interestingly, most molecular studies focus solely on adults, leaving fetal discs, which have abundant notochordal cells relatively undiscovered. Since fetal discs undergo rapid growth, their ECM proteome contain many proteins involved in anabolic pathways. Identification of these proteins could give rise to new insights in the pathophysiology and regenerative treatment strategies which revolve around the restorement of ECM in both quantity and quality.

Unfortunately, very little is known regarding the ECM of the human fetal NP**.** The studies that have been conducted in this field, focused on developmental structures and changes of specific ECM proteins^14–17^. In addition, Caldeira et al., (2017) have compared the proteome between bovine- fetal and adult discs^18^. Until now, no studies have been conducted that performed a qualitative and quantitative exploration of the human fetal matrisome (ECM proteins).

Hence, the aim of this explorative study is to identify proteins of interest for future therapies through profiling the core matrisomal proteome of human fetal NP’s, and compare it with the core matrisome of healthy adults.

## Materials and methods

### Patient population

The main focus of this study is to compare two groups: Fetal and healthy adult NP’s. Fetal NP tissue was harvested from 9 miscarriages from all lumbar and thoracic spinal segments. Healthy adult NP tissues were harvested from brain dead organ donor volunteers, of which 2 discs were harvested from L3 to L4 and 5 discs from L4 to L5. Moreover, 11 degenerated discs were included for an additional analysis, 1 originated from the level L2L3, 1 from L3L4, 7 from L4L5 and 2 from L5S1. Pfirmann scores of all included NP’s are listed in Supplementary Table S1. Institutional Ethical Committee (Ganga hospital Ganga Medical Centre and Hospital Pvt Ltd Tamil Nadu) and Institutional Review Board approval were obtained to conduct research on these specimens. Informed consent was obtained for each included sample and all methods were carried out according to the Declaration of Helsinki.

### Sample collection

Fetal specimens were gathered following spontaneous as well as medical abortions (24 weeks of gestational age). The spine segment was harvested through a posterior approach within 12 h of death under sterile operating conditions after obtaining an informed consent from the parents of the deceased fetus. Nine Fetal samples with normal MRI and CT were included and those with congenital anomalies were excluded from the study. Intervertebral disc tissues were dissected and subsequently separated into nucleus pulposus and annulus fibrosus using a surgical microscope.

Regarding the healthy adult NP tissues, our institution has been licensed by the government for harvesting bone through a voluntary organ donor program. The spine was procured as 2/3 motion segments after retrieval of organs such as kidneys, heart and liver for transplant surgeries. MRI was obtained and NP’s were harvested after segregating bone and annulus fibrosus. Both Fetus and adult NP’s were transferred to sterile cryopreservation vials, and snap frozen in liquid nitrogen before transport to the research laboratory.

### Sample processing

Around 200 mg tissue from the 7 adult, 9 fetus and 11 degenerated discs was subjected for extraction of total proteins and tryptic digested peptides were subjected to ESI-LC–MS/MS with conditions as described in our earlier report: (Rajasekaran et al.^19^).

### Bioinformatics analysis

MS/MS raw data acquired from Orbitrap Velos Pro Mass Spectrometer were analysed by Proteome Discoverer v1.4 using Mascot (Matrix Science, London, UK; version 2.4.1.0) and inbuilt SequestHT search algorithm. The peptide spectrum matches (PSMs) from SequestHT and Mascot were post-processed using the Percolator algorithm. The peptides with rank one and having a q-value < 0.01 were considered for protein identification.

### Relative quantification by spectral count

Spectral counts obtained by LC/MS–MS were further normalization by normalized spectral abundance factor (NSAF) method, as described by Zybailov et al.^20^. NSAF is a relative quantification method based on protein length and spectral count. NSAF is calculated as follows:

$$ (NSAF)k = \frac{{\left( \frac{SpC}{L} \right)k}}{{\sum\nolimits_{i = 1}^{N} {\left( \frac{SpC}{L} \right)i} }} $$

SpC: Number of spectral counts; L: Protein Length; k: individual protein.

### Quantitative analysis

Out of the proteomic database, the core matrisome (ECM proteoglycans, glycoproteins and collagens) was selected for further analysis^21^. All selected proteins with > 2 unique peptide or 1 unique peptide with a PSM ≥ 10 were included in the analysis^22^.

### Statistical analysis

Data was analyzed using SPSS software version 25. Since the sample size was limited, differences in protein expression between groups were assessed using Mann–Whitney *U* tests. Groups were only compared if more than two samples expressed the respective protein in each group. Two-tailed alfa level was set at 0.05. Samples with missing values were excluded from the analysis.

### Pathway analysis

In order to identify fetus specific proteins of interest for future regenerative treatment, a pathway analysis of all fetal specific proteins was performed using both STRING v10.5 and DAVID v.6.8 databases. Proteins were considered ‘fetus specific’ if they met one of the following three conditions: Firstly, they were uniquely described in fetal NP’s in at least 2 fetal samples. Secondly, they were significantly upregulated in fetal NP’s. Thirdly, in the case that only one sample was identified in the healthy adult group and no statistical analysis could be performed, an upregulation in fetal expression of at least a twofold.

### Interaction analysis

In order to find out whether the identified pathways interact and share common initiators, a protein–protein interaction analysis was performed using Cytoscape v3.7^23^.

### Verifying in degenerated samples

In order to assess whether the protein that decrease from fetus to healthy adult NP’s further decrease in severely degenerated discs, expression levels of all proteins of interest that were identified in regenerative pathways were used for an additional analysis. In this analysis, protein expression in 11 degenerated discs was compared to expression levels in fetus and healthy adult NP’s.

### Validation by immunohistochemistry

Immunohistochemistry (IHC) was used to validate proteomic data: expression differences between the groups were assessed for three proteins: Collagen 14A1, Fibromodulin and Biglycan. For Collagen 14A1 and Biglycan 4 fetus NP’s (from one fetal spine) and 4 adult NP’s from different organ donors were used. For Fibromodulin, two samples were used per group (all from different donors). Samples were fixed in formalin solution and subsequently embedded in paraffin. 5 μm section from the paraffin blocks were used for IHC using a tree-step indirect method. For antigen retrieval, samples were heated in retrieval buffer until 15 lb per square inch pressure was reached, which was subsequently maintained for 2 min. Slices were rinsed in ethanol solutions and incubated in 0.1% hydrogen peroxide for blocking. Monoclonal antibodies for Fibromodulin (Santa Cruz Biotechnology, Inc USA) and Collagen14a1 (Santa Cruz Biotechnology, Inc USA), Biglycan (Santa Cruz Biotechnology, Inc USA), were used. Primary anti-bodies were incubated for 1 h and secondary antibodies (1:100 dilution) were developed with DAB and counterstained with Harris hematoxylin. Images were captured using Leica Application Suite Vs 4.5.0.418 software for a Leica DML light microscope. Evaluation of the slides was performed by an experienced pathologist. Slides were scored as negative, or as a mild/moderate/strong positive.

## Results

### Descriptive analysis of ECM

#### Gene ontology

**Figure 1:**
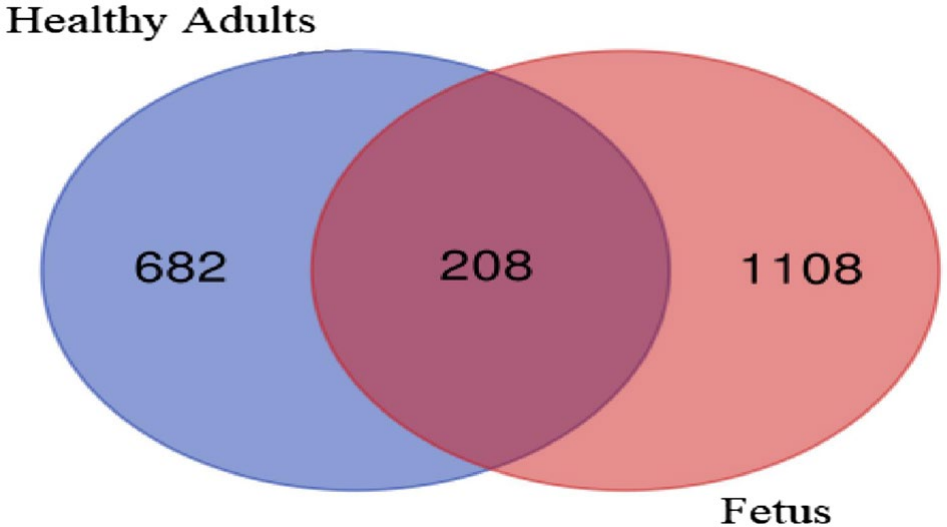
Venn diagram representing total number of identified proteins expressed in fetal and healthy adult NP’s: Blue displays the unique number for healthy adults, Red the unique number for fetus, and Purple the number of share proteins.

In fetal NP’s, a total of 1316 different proteins were identified, of which 1108 were unique to fetus and 208 were shared with adult NP’s. In adult NP’s, a total of 889 different proteins were identified, of which 682 unique to adult NP’s (Fig. 1).

The quantitative analysis revealed that 48% of the fetal NP proteome consisted of ECM proteins (core matrisome + regulators + ECM affiliated + secreted factors), the remaining 52% was characterized as cellular. For healthy adults, the percentage of ECM proteins was 64%.

The matrisome consisted of 86% of core matrisomal proteins in fetal NP’s, similar results were seen in adult discs (90%). The core matrisome was subdivided in proteoglycans (56% in fetus and 82% in adult), glycoproteins (24% in fetus and 17% in adult) and collagens (20% in fetus and 2% in adult).

**Figure 2:**
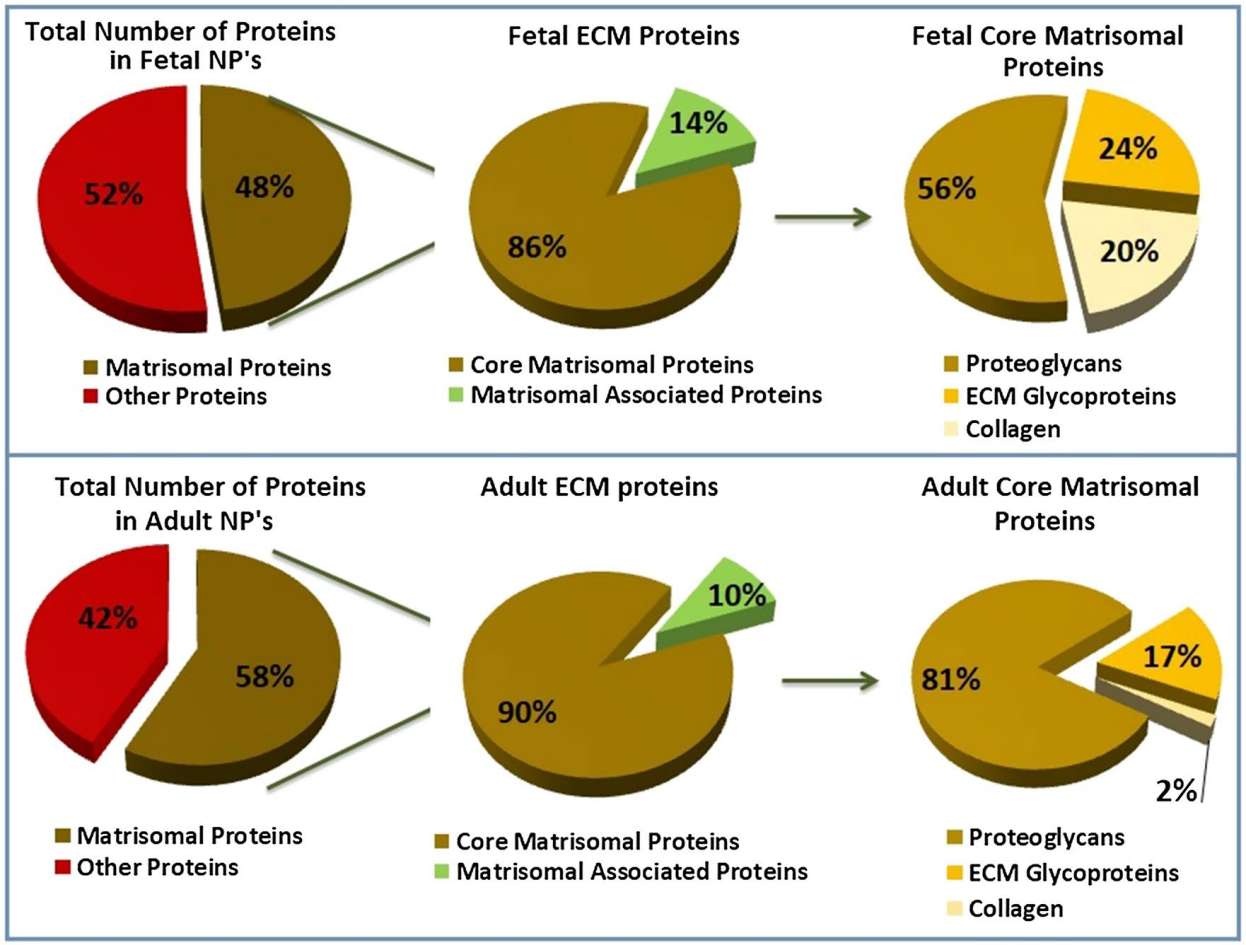
Relative protein composition fetus and healthy adult NP’s: the pie chart exhibiting percentages of identified proteins distributed by matrisomal and other proteins.

The composition of the discs is illustrated in Fig. 2.

### Comparing fetal and adult core matrisome

**Figure 3:**
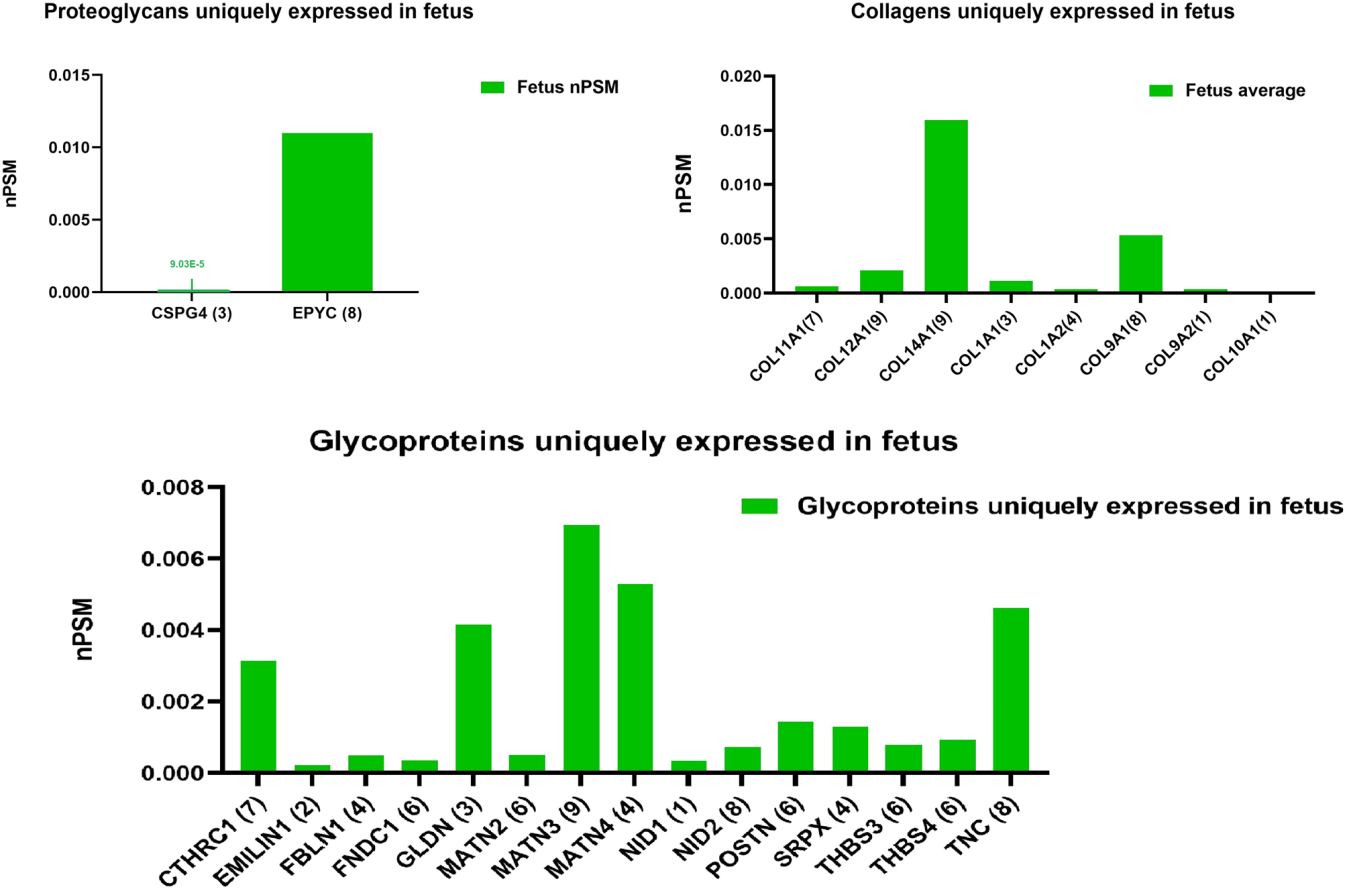
Proteins that were uniquely expressed in fetal NP’s: the Y axis displays median normalized PSM’s per group, X axis shows protein gene symbols accompanied by (number of samples). The upper left graph displays proteoglycans, the upper right collagens and the lower graph glycoproteins.

After applying the exclusion criteria, a total of 64 core matrisomal proteins were subjected for further analysis. Out Of these 64, 25 proteins were specific to fetus, of which 2 proteoglycans, 15 glycoproteins, and 8 collagen types (Fig. 3, Supplementary Table 2). In adult NP’s only 6 proteins were specific and belonged to glycoproteins (Supplementary Table 2).

**Figure 4:**
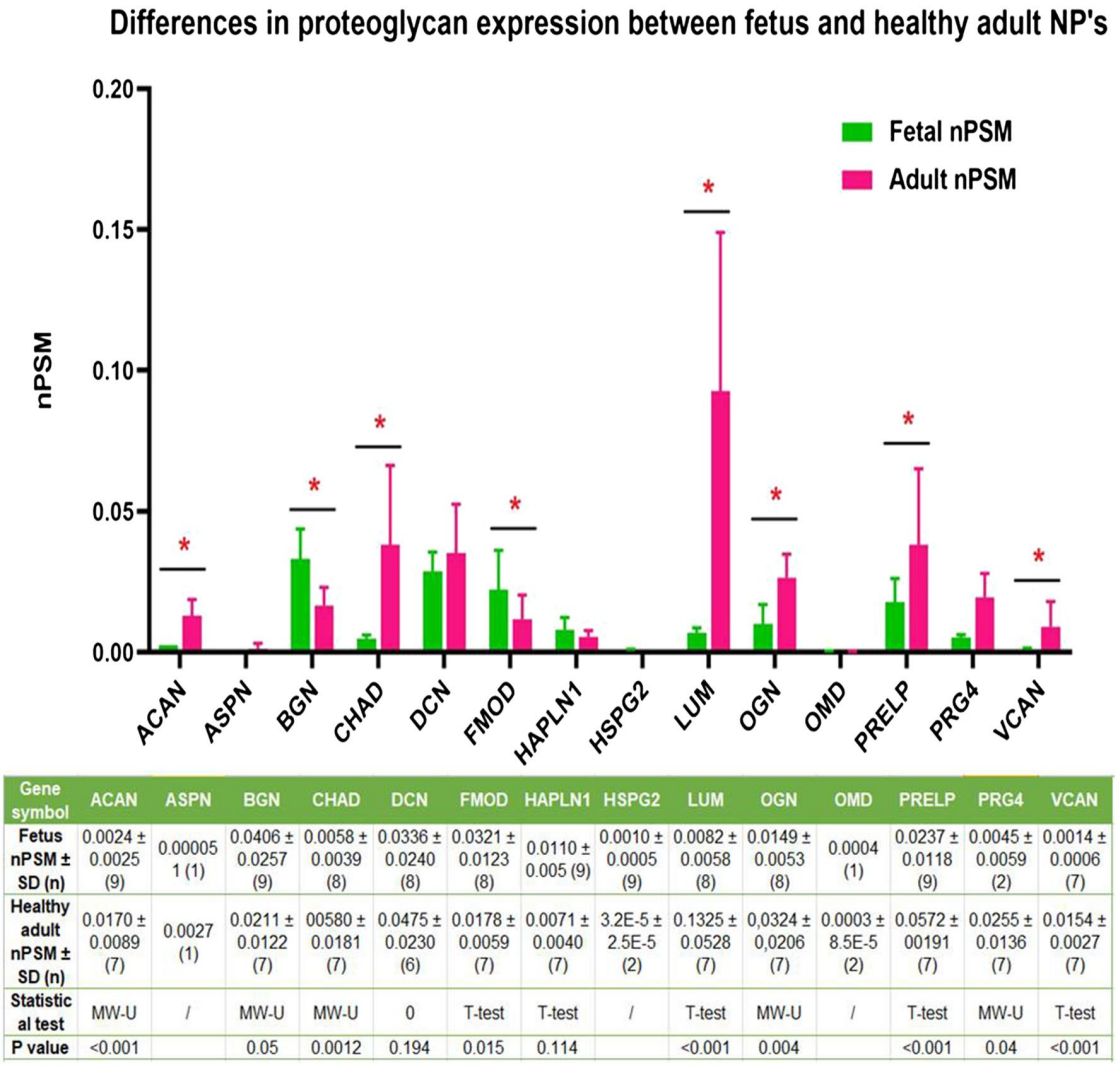
Differences in proteoglycan expression between fetus and healthy adults: The Y axis displays median normalized PSM’s per group, X axis shows protein gene symbols, *indicates statistical significance and error bars are Interquartile ranges, which were only displayed if n > 2. The attached table contains the median (number of samples) for each group, and provides the according Mann Whitney U p-values if statistics could be performed (n > 2). ‘/’ indicates that only 1 or 2 samples were present in one of the groups and no statistical test could be performed.

Furthermore, 22 out of the 64 proteins were expressed in > 2 samples in each group and were selected for statistical analysis. The analysis for proteoglycans revealed a significantly lower expression of aggrecan (p = 0.001), chondroadherin (p = 0.001), lumican (p = 0.001), osteoglycin (p = 0.004), ‘proline and arginine rich end leucine rich repeat protein’ (PRELP) (p = 0.004) and versican (p = 0.001), and significantly higher expression of biglycan (p = 0.05) and fibromodulin (p = 0.028) in fetus (Fig. 4).

**Figure 5:**
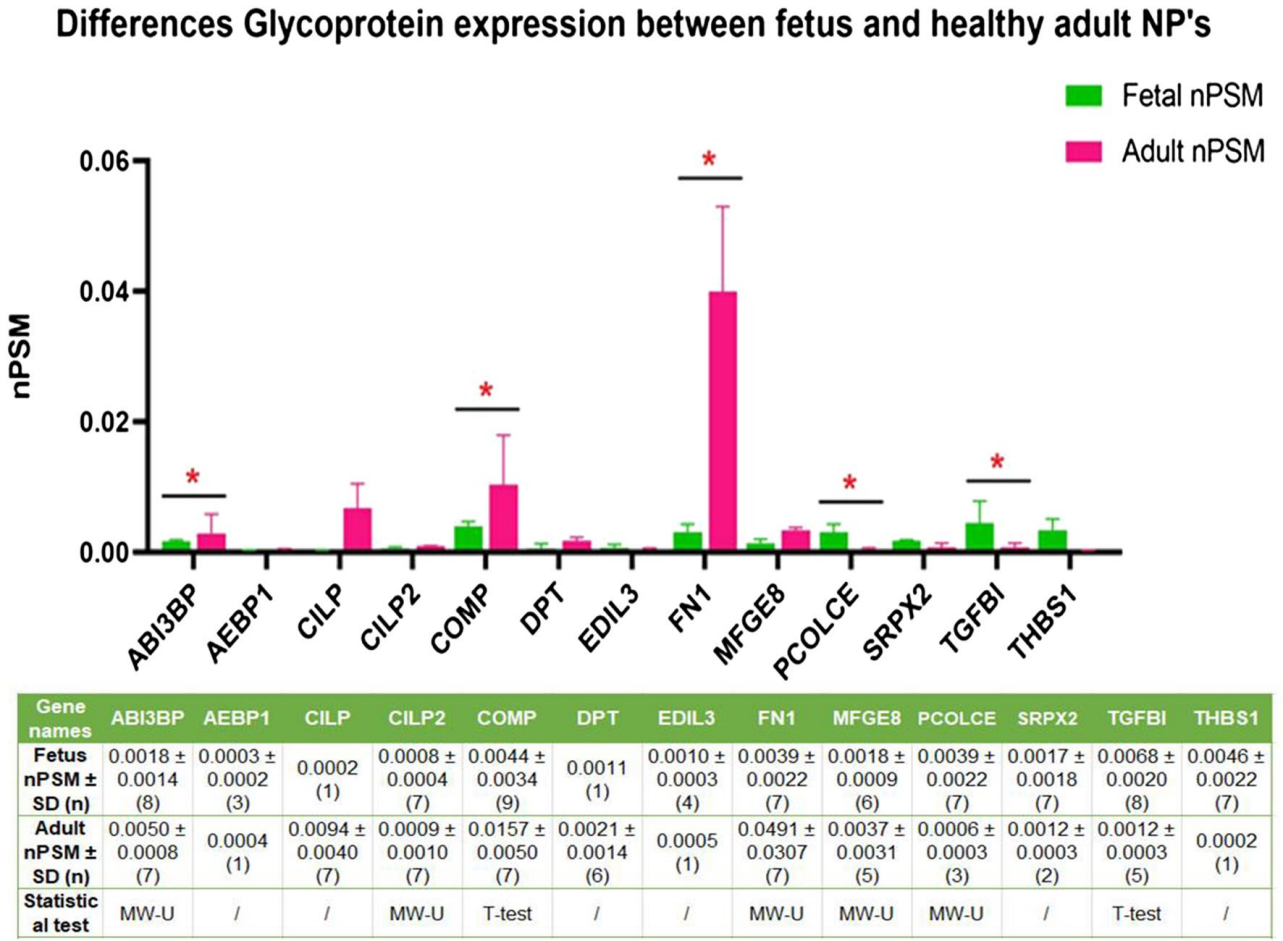
Differences in glycoprotein expression between fetus and healthy adults: The Y axis displays median normalized PSM’s per group, X axis shows protein gene symbols, error bars are Interquartile ranges and * indicates statistical significance. The attached table contains the median (number of samples) for each group, and provides the according Mann Whitney U p-values if statistics could be performed (n > 2). ‘/’ indicates that only 1 or 2 samples were present in one of the groups and no statistical test could be performed.

Regarding the glycoproteins, significant downregulations in fetal NP’s as compared to adult NP’s were found for ‘ABI family, member 3 (NESH) binding protein’ (ABI3BP) (p = 0.004), ‘cartilage oligomeric matrix protein’ (COMP) (p = 0.001) and fibronectin (FN1) (p = 0.002). In contrast, ‘procollagen C-endopeptidase enhancer (PCOLCE)’ (p = 0.02) and ‘transforming growth factor-beta-induced protein ig-h3 (TGFBI) (p = 0.003) were both significantly upregulated in fetal NP’s (Fig. 5).

**Figure 6:**
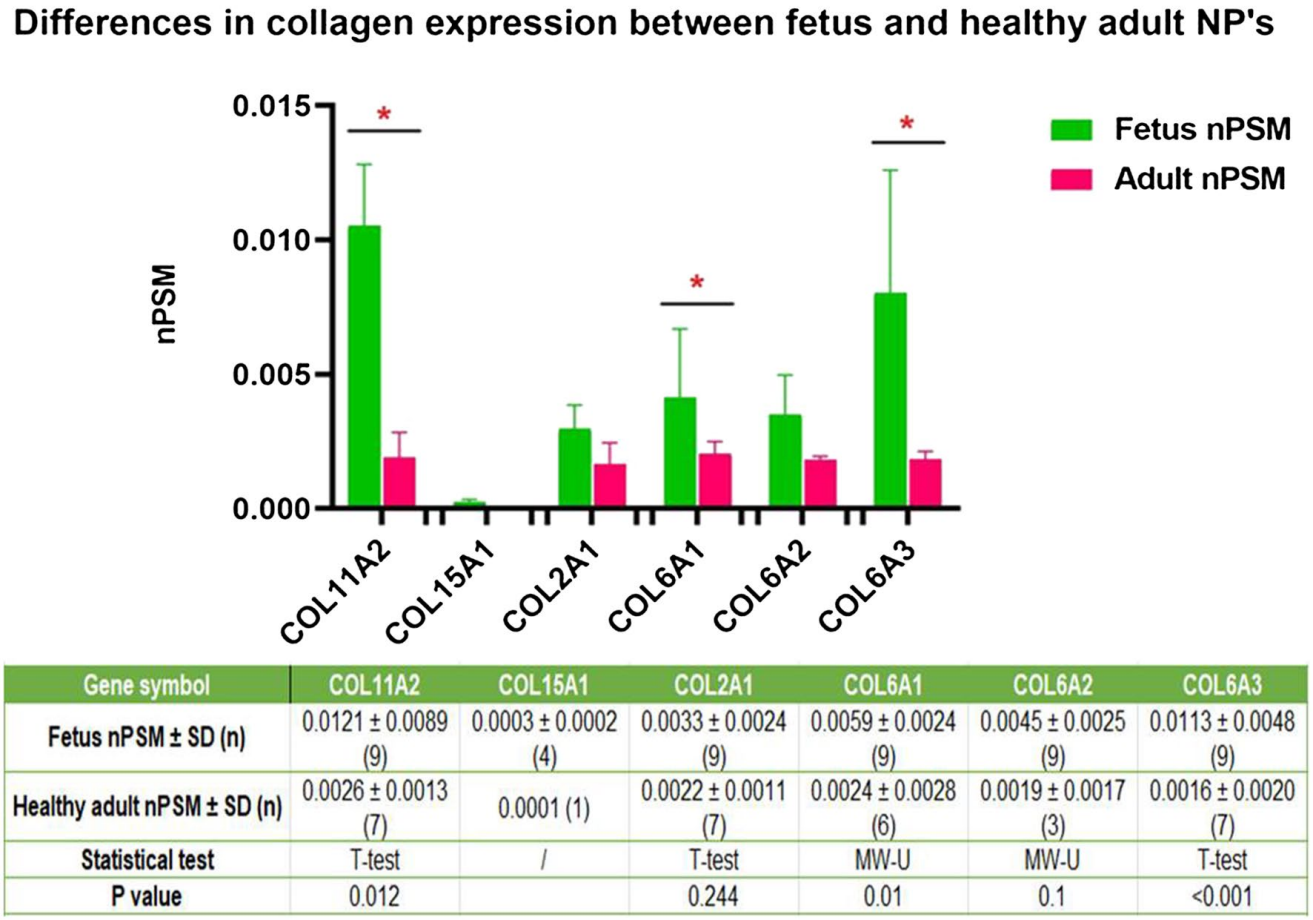
Differences in collagen expression between fetus and healthy adults: The Y axis displays median normalized PSM’s per group, X axis shows protein gene symbols, error bars are Interquartile ranges and * indicates statistical significance. The attached table contains the median (number of samples) for each group, and provides the according Mann Whitney U p-values if statistics could be performed (n > 2). ‘/’ indicates that only 1 or 2 samples were present in one of the groups and no statistical test could be performed.

At last, comparing fetal and adult collagen expression showed that collagen types 11a2 (COL11a2) (p = 0.012), COL6a1 (p = 0.05) and COL6a3 (p < 0.001) were significantly upregulated in fetal NP’s (Fig. 6).

Moreover, the remaining 11 proteins were present in both groups but were not eligible for statistical analysis (< 3 samples in one of the groups). Of those proteins, the proteoglycan ‘Heparan sulfate proteoglycan 2’ (HSPG2), the glycoproteins ‘EGF-like repeats and discoidin I-like domains 3 (EDIL3)’ and thrombospondin 1 (THBS1), and collagen type 15A1 (COL15A1) were upregulated > twofold in fetus samples. Hence these proteins were also integrated in the pathway analysis.

### Identifying fetal proteins of interest for regenerative potential

Based on the previously described results, a total of 5 proteoglycans, 18 glycoproteins and 10 collagen types were selected (Table 1). STRING database provided a total of 56 pathways in which these proteins were involved. Out of these pathways, 7 were involved in synthesis of the extracellular matrix and 2 in growth related pathways (Table 2). Moreover the 33 selected proteins were involved in 90 biological pathways, out of which 4 were growth related. All selected collagen types, biglycan, fibromodulin, HSPG2, ‘PCOLCE’ and ‘Chondroitin sulfate proteoglycan 4 (CSPG4)’ were involved in ECM assembly pathways. Regarding the growth-related pathways, collagen types 1A1, 1A2, 6A1,6A1, 9A1, 11A1, and 11A2, thrombospondin 1, 3 and 4, matrilin 3 and tenascin C were identified (Table 2). Concerning the biological processes, collagen 1A1, 6A1, 11A1, 11A2 and 12A1, Collagen triple helix repeat containing 1 (CTHRC1), periostin and TGFBI, thrombospondin 3 and tenascin C were involved in ‘tissue development’. Additionally, collagen 1A1, 1A2, fibromodulin, periostin and thrombospondin 1 are involved in ‘cellular response to growth factor stimulus’. The last relevant pathway was ‘regeneration’, in which matrilin-2, periostin and tenascin C were present.

**Table 1:** Protein list of interest.

Protein name	Gene symbol	Cluster
Collagen triple helix repeat containing 1	CTHRC1	Glycoprotein
EGF-like repeats and discoidin I-like domains 3	EDIL3	Glycoprotein
Elastin microfibril interfacer 1	EMILIN1	Glycoprotein
Fibulins	FBLN1	Glycoprotein
Fibronectin type III domain containing 1	FNDC1	Glycoprotein
Gliomedin	GLDN	Glycoprotein
Isoform 3 of matrilin-2	MATN2	Glycoprotein
Isoform 2 of matrilin-3	MATN3	Glycoprotein
Matrilin-4	MATN4	Glycoprotein
Nidogen 2	NID2	Glycoprotein
Procollagen C-endopeptidase enhancer	PCOLCE	Glycoprotein
Periostin, osteoblast specific factor	POSTN	Glycoprotein
Sushi-repeat-containing protein, X-linked	SRPX	Glycoprotein
Transforming growth factor-beta-induced protein ig-h3	TGF-BI	Glycoprotein
Thrombospondin 1	THBS1	Glycoprotein
Thrombospondin 3	THBS3	Glycoprotein
Thrombospondin 4	THBS4	Glycoprotein
Tenascins	TNC	Glycoprotein
Biglycan	BGN	Proteoglycan
Chondroitin sulfate proteoglycan 4	CSPG4	Proteoglycan
Epiphycan	EPYC	Proteoglycan
Fibromodulin	FMOD	Proteoglycan
Heparan sulfate proteoglycan	HSPG2	Proteoglycan
Collagen, type I, alpha 1	COL1A1	Collagen
Collagen, type I, alpha 2	COL1A2	Collagen
Collagen, type VI, alpha 1	COL6A1	Collagen
Collagen, type VI, alpha 3	COL6A3	Collagen
Collagen, type IX, alpha 1	COL9A1	Collagen
Collagen, type XI, alpha 1	COL11A1	Collagen
Collagen, type XI, alpha 2	COL11A2	Collagen
Collagen, type XII, alpha 1	COL12A1	Collagen
Collagen, type XIV, alpha 1	COL14A1	Collagen
Collagen, type XV, alpha 1	COL15A1	Collagen

Table lists the proteins of interest (uniquely expressed in fetus in at least 2 samples, significantly upregulated in fetus or upregulated > twofold if only 1 sample expressed the respective protein in the healthy adult group).

**Table 2:** Pathway analysis.

	Proteins involved	P-value	Gene symbol
**ECM assembly pathways**			
Assembly of collagen fibrils and other multimeric structures	11	3.72e−17	COL11A1, COL11A2, COL12A1, COL14A1, COL15A1, COL1A1, COL1A2, COL6A1, COL6A3, COL9A1, PCOLCE
Collagen biosynthesis and modifying enzymes	11	7.47e−17	COL11A1, COL11A2, COL12A1, COL14A1, COL15A1, COL1A1, COL1A2, COL6A1, COL6A3, COL9A1, PCOLCE
Crosslinking of collagen fibrils	3	5.19e−05	COL1A1, COL1A2, PCOLCE
Glycosaminoglycan metabolism	4	0.00043	BGN, CSPG4, FMOD, HSPG2
Anchoring fibril formation	2	0.0015	COL1A1, COL1A2
Chondroitin sulfate biosynthesis	2	0.0021	BGN, CSPG4
**Growth pathways**			
PI3K-Akt signalling pathway	9	1.89e−08	COL1A1, COL1A2, COL6A1, COL6A3, COL9A1, THBS1, THBS3, THBS4, TNC
Regulation of Insulin-like Growth Factor (IGF) transport and uptake by Insulin-like Growth Factor Binding Proteins (IGFBPs)	2	0.0046	MATN3, TNC
**Biological processes**			
Tissue development	10	0.0043	COL11A1, COL11A2, COL12A1, COL1A1, COL6A1, CTHRC1, POSTN, TGFBI, THBS3, TNC
Cellular response to growth factor stimulus	5	0.0128	COL1A1, COL1A2, FMOD, POSTN, THBS1
Regeneration	3	0.0197	MATN2, POSTN, TNC

Table displays the results of the STRING and DAVID pathway analysis. The first (left) column shows the identified pathways and biological processes that are involved in ECM assembly, the second column shows the number of proteins of interest involved in that pathway/process, the third list the p-value of the upregulation and the fourth displays the gene symbols of the proteins involved. In total, 24 proteins were identified in 8 pathways and 3 biological processes.

### Interaction analysis

Protein–protein interaction analysis by Cytoscape revealed that biglycan has outgoing interactions to proteins in all the relevant ECM assembly pathways, and the P13K-Akt-pathway (Fig. S1). In addition, biglycan received incoming interactions from fibromodulin, TGFBI and HSPG1. Most importantly, it also received input from thrombospondin 4, matrilin-2, matrilin-3 and periostin. The interactions between these proteins connect the IGF-signaling pathway with some of tissue remodeling, cellular response to growth factors and regeneration processes through biglycan signaling (Fig. S2).

### Verification in degenerated samples

**Figure 7:**
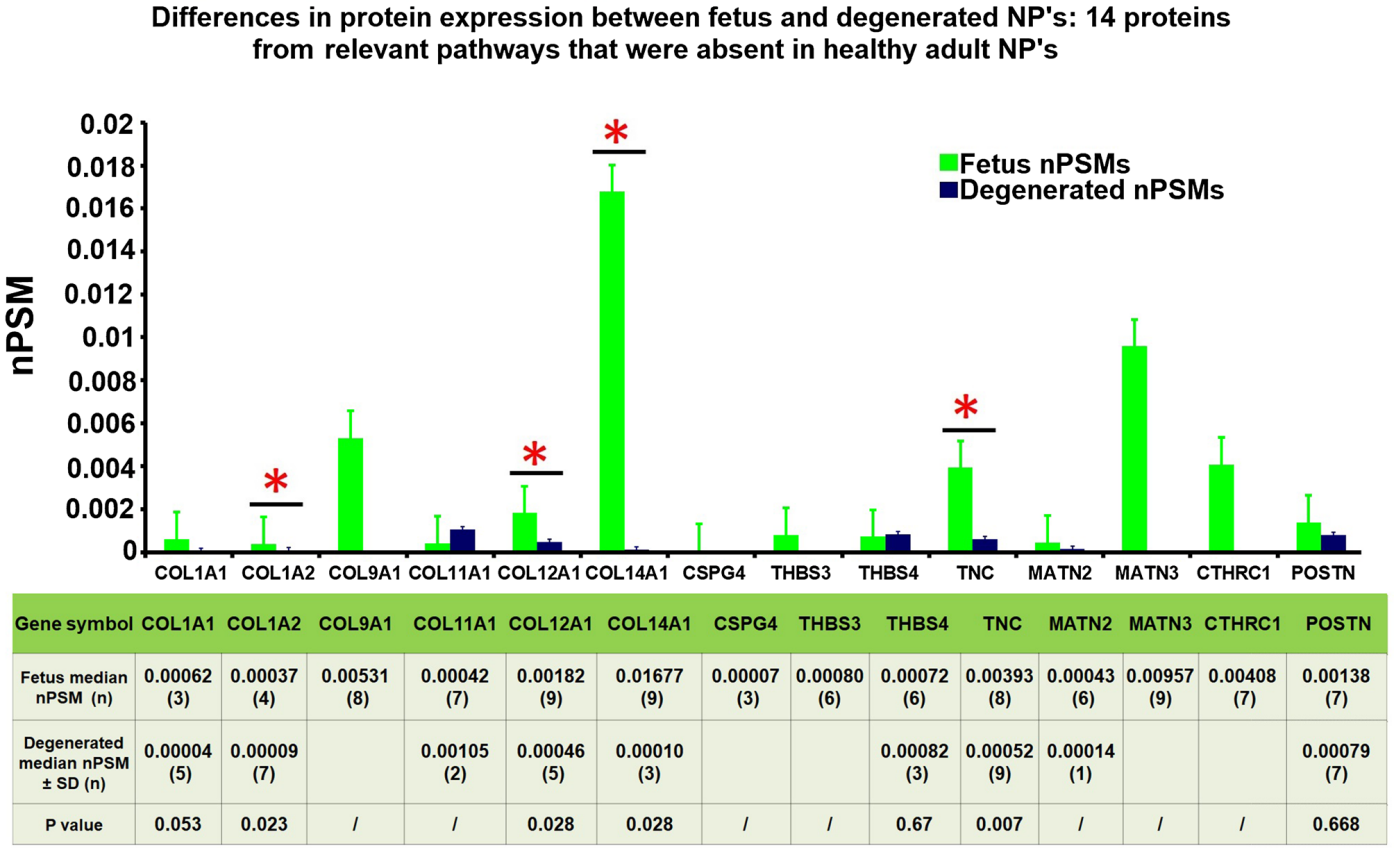
Differences in protein expression between fetus and degenerated NP's: 10 proteins from relevant pathways that were present in healthy adult NP's. The Y axis displays median normalized PSM’s per group, X axis shows protein gene symbols, error bars are Interquartile ranges and *indicates statistical significance. The attached table contains the median (number of samples) for each group, and provides the according Mann Whitney U p-values if statistics could be performed (n > 2). ‘/’ indicates that only 1 or 2 samples were present in one of the groups and no statistical test could be performed.

The 24 proteins selected in the relevant regenerative pathways were submitted to an additional analyses, in which the expression levels in adult NP was compared to degenerated NP’s. 14 out of the 24 proteins were uniquely expressed in fetal discs and absent in adult discs, in line with the decrease from fetus to adult, 5 proteins were also absent in degenerated discs: Collagen 9a1, CSPG4, Matrillin-3, thrombospondin 3 and CTHRC1. In addition, Matrillin-2 was expressed in only 1- and Collagen 11a1 in 2 degenerated samples. By contrast, 7 proteins that were absent in healthy adult discs were present in degenerated discs: Collagen 1a1, 1a2, 12A1, 14A1, tenascin, thrombospondin 4 and periostin (Fig. 7).

**Figure 8:**
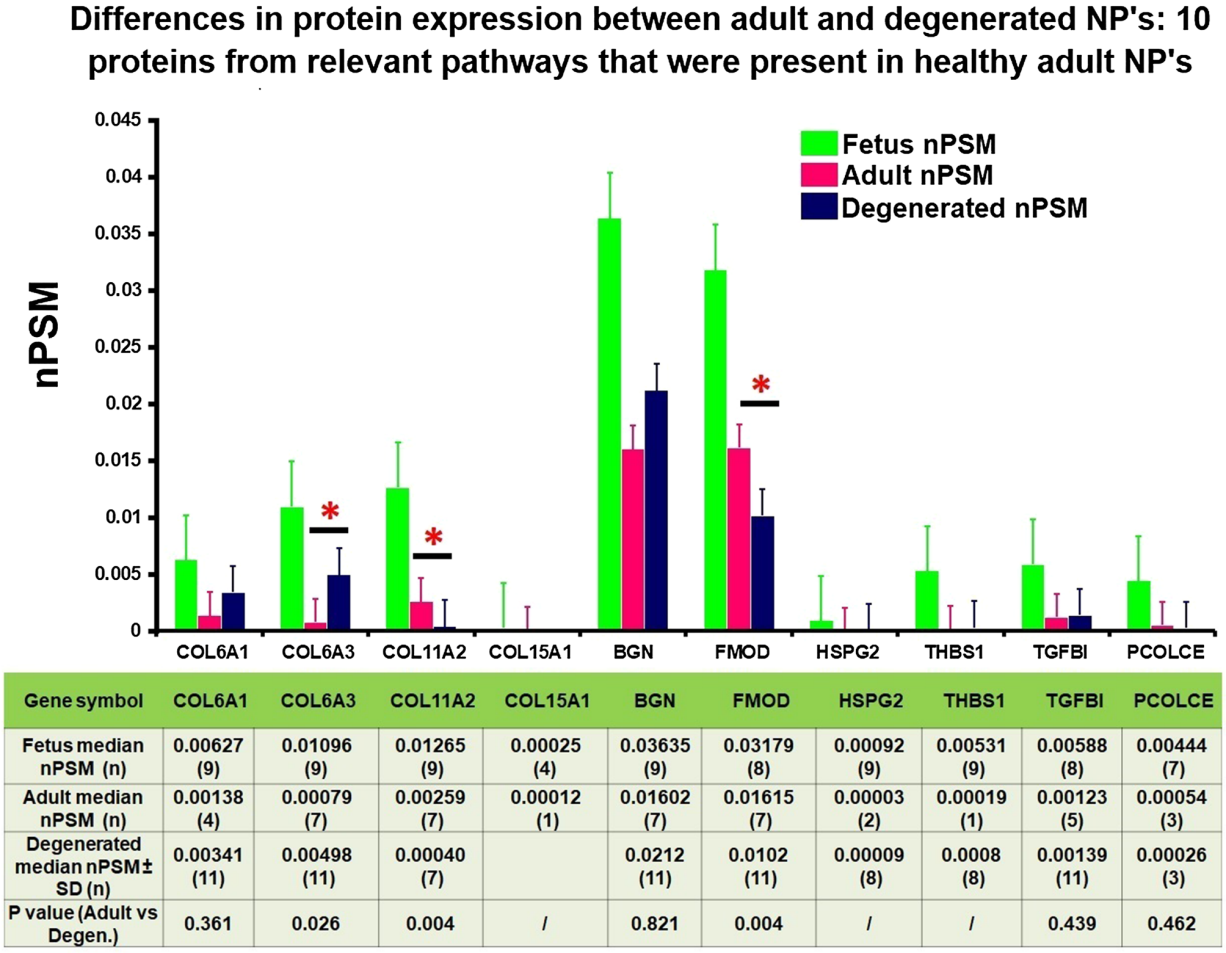
Differences in protein expression between healthy adult and degenerated NP's: 10 proteins from relevant pathways that were present in healthy adult NP's. The Y axis displays median normalized PSM’s per group, X axis shows protein gene symbols, error bars are Interquartile ranges and *indicates statistical significance between healthy and degenerated Adults. The attached table contains the median (number of samples) for each group, and provides the according Mann Whitney U p-values if statistics could be performed (n > 2). ‘/’ indicates that only 1 or 2 samples were present in one of the groups and no statistical test could be performed.

Of the 10 proteins that were expressed in both fetus and healthy adult NP’s, Collagen 11A2 and Fibromodulin showed a significantly lower expression in degenerated compared to healthy adult NP’s, which was in line with the decrease from fetus to healthy adult. In contrast, collagen 6a3 was upregulated in degenerated compared to healthy adult NP’s. The other 8 proteins showed no significant differences (Fig. 8). In short, of the 24 relevant proteins that decreased in healthy adults compared to fetus, 8 proteins increased again upon degeneration.

### Validation by immunohistochemistry

**Figure 9:**
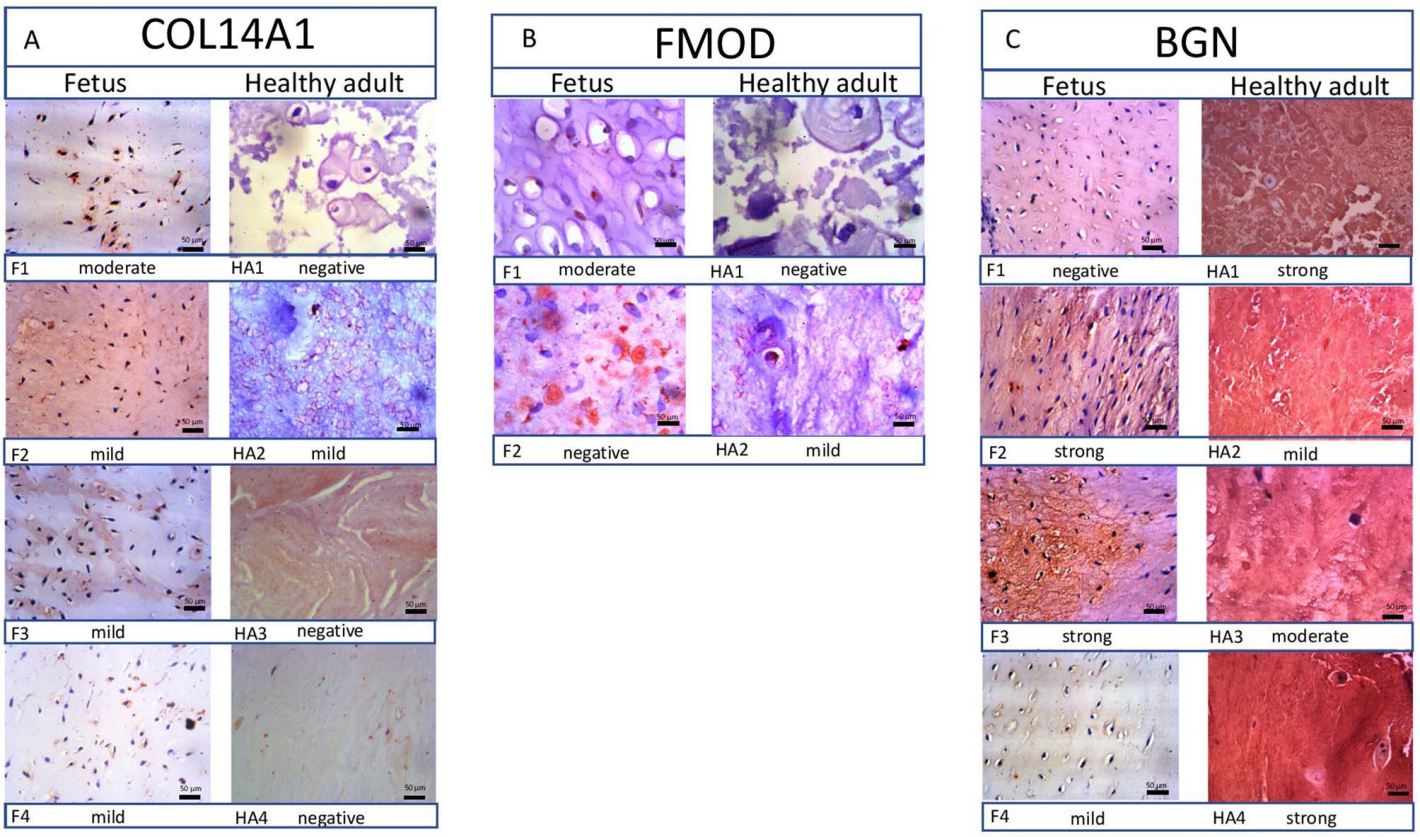
Validation by immunohistochemistry: protein expression of Col14a1, FMOD, and BGN was validated by immunohistochemistry (magnification 400 ×, scale bar = 50 µm). (**A**) COL14a1 was strongly positive in the interstitium and cell margins of fetus NP’s (F), Healthy adult (HA) samples, faint staining was seen in the interstitium of HA2. (**B**) FMOD was moderately positive in the cell margins and interstitium of F1, but negatively stained in the rest of the NP’s. In HA, only sample HA2 was stained mildly positive. (**C**) BGN was stained positively in all HA and all but one F NP with varying intensity. In HA, the staining was diffuse in the interstitium but not in the NP cells, whereas in F NP’s the staining was positive in cytoplasm and interstitium.

4 Fetus samples and 4 adult control discs underwent immunohistochemistry for validation. Protein expression of COL14a1, FMOD, and biglycan was validated by IHC. COL14A1 was present in all fetus NP’s with mild or moderate intensity, and present in only one adult NP with mild intensity (Fig. 9A). Both fetus NP samples showed a strong positive FMOD staining, whereas in adult NP’s only 1 sample was moderately positive whereas the other was negative. Together, these results validate the proteomic findings (Fig. 9B). Biglycan was strongly positive in 3 out of the 4 fetus samples, and mild to strongly positive in 4 adult samples. Thereby verifying their abundant presence in both, without any clear differences in quantity. The staining pattern in adults was completely diffuse, whereas in fetus it was centered around the cells (Fig. 9C).

## Discussion

The fetal NP proteome is highly different from that of a healthy adult. Using a proteomic approach combined with a bioinformatics pathway analysis, this study identified significant upregulations or unique expressions in fetal NP’s of 33 core matrisomal proteins, of which 24 involved ECM assembly or growth pathways. Of these 24, 2 proteins further decreased in degenerated discs, whereas 8 increased. Moreover, this study is the first to describe the core matrisomal proteome of human fetal NP’s.

Our findings are in line with those of Smith et al.^17^, who performed a immunohistochemical analysis of multiple proteoglycan and collagen types in the human fetal disc. They also found a low expression of aggrecan, versican and perlecan (a synonym for HSPG2) in the intervertebral disc. In addition, they reported on the presence of both collagen type 1 and 2 in the nucleus pulposus, which was confirmed by our results. Collagen type 1 is known for its presence in AF but not in NP tissue. Their presence in fetus NP’s could indicate that the distinguishment between AF and NP regions is not yet fully developed at this stage.

When comparing our results with proteomic data from bovine discs as described by Caldeira et al.^18^, we found the overall distribution of the matrisome to be quite comparable. When comparing fetus and healthy adult for significant differences, similar results in terms of expression were found for chondroadherin, lumican, PRELP, cartilage oligomeric matrix protein and Fibronectin. However, some results were different; in bovine adult discs, the expression of fibromodulin and ‘Hyaluronan and proteoglycan link protein 1’ was found higher as compared to fetal discs, which contradicted the differences in expression levels of human discs. Nevertheless, it should be noted that the higher expression in human fetus was only significant for fibromodulin. In order to verify whether these differences can be attributed to interspecies variation or to unsystematic error due to small sample sizes, more proteomic studies on fetal discs are needed. In addition, Bach et al. performed a qualitative proteomic study using notochordal cell conditioned medium from humans, canine and porcine^24^. Their results were comparable with expression profile of fetus NP’S: apart from heparan sulfate proteoglycan, periostin and Collagen triple helix repeat containing 1, all identified proteins of interest were expressed in their medium.

### Differences between healthy and degenerated adult discs

The additional analysis with degenerated discs revealed that of the 24 identified relevant proteins, only fibromodulin and collagen11a2 further decreased in degenerated discs compared to healthy adults. This places additional interest on these 2 proteins as potential targets for regenerative therapy. By contrast, even though in low concentrations and in mostly limited amount of samples, 9 proteins that were absent in adult discs were re-expressed in the degenerated state and one showed significantly increased expression compared to healthy adult discs (Collagen 1a1, 1a2, 6a3, 11a1, 12A1, 14A1, Matrillin-2 tenascin, thrombospondin 4 and periostin). This could mean that these proteins are involved in inducing the degenerated state, for example through functioning as an inhibitor or regulator in the regenerative processes identified. By contrast, these proteins could also be upregulated as a compensation mechanism. If this is the case, further increasing their concentrations may aid regeneration and slow further decay. In order to distinguish between harmful and compensatory, mechanical experiments are required, which is beyond the scope of this study. However, in order to identify the best candidates for future mechanical experiments, we will elaborate on the specific functions on all 24 identified proteins below.

### ECM assembly pathways: proteoglycans

Regarding the proteoglycans, only Fibromodulin, CSPG4, HSPG2 and biglycan showed up in relevant ECM growth related pathways. All four were involved in ‘glycosaminoglycan metabolism’, which support fibrils and control hydrostatic pressure^25^. CSPG4 is a glycosaminoglycan with anti-inflammatory, anti-catabolic to anti-apoptotic potential, which is currently used as a treatment for osteoarthrosis of the knee joint^26^. Furthermore, recent literature revealed that biglycan and fibromodulin are involved in both the assembly and regulation of collagen fibrilogenesis^9^. Interestingly, biglycan was also identified as an initiator for multiple pathways in the interaction analysis. It is a cell-associated class- I SLRPs and is secreted as probiglycan which is cleaved by the BMP-1 to form mature biglycan^27^. Secreted biglycan interacts with collagen type I, II, III, and VI and elastin and becomes sequestered in the ECM of most of the organs^28^, In the developing human fetal discs, biglycan is widely distributed and its presence separates the interspace of the developing intervertebral disc from that of the cartilage end plate. Moreover, biglycan also has various interactions with growth factors and receptors, and through these interactions it acts as a signaling molecule that regulates cell growth and morphogenesis^29^.

Even though these proteins display regenerative potential, it should be noted that the p-values of the differences in expressions of HSPG2, biglycan and Fibromodulin were > 0.01, and that this study has performed multiple tests without corrections for multiple testing, hence the findings should be interpreted with caution. Similar caution in interpretation is required regarding the uniquely expressed CSPG4, since it was only present in 3 out of the 8 samples. Nevertheless, the difference in expression of FMOD was validated by IHC. Besides, based on their functions, all three seem to be potential candidates for regenerative therapies. Interestingly, the IHC data revealed that in fetus NP’s fibromodulin was solely located in the nuclear membrane, whereas in adult NP’s it was spread over the cell cytoplasm and interstitium. This illustrates that at 24 weeks; FMOD is not yet part of the extracellular matrix and still has to be excreted to fulfill its role in collagen fibrillogenesis.

### ECM assembly pathways: glycoproteins

Out of the 18 relevant glycoproteins, PCOLCE was involved in 4 relevant ECM growth related pathways: ‘Assembly of collagen fibrils and other multimeric structures’, ‘Collagen biosynthesis and modifying enzymes’ and ‘Collagen formation’ and ‘Crosslinking of collagen fibrils’. This enzyme is necessary for the conversion of procollagen to collagen (type I–III) and is thereby crucial in the creating of a fibrillary matrix^30^.

### ECM assembly pathways: collagens

Interestingly, all upregulated or unique fetus collagen types were involved in ECM assembly pathways: Assembly, anchoring and crosslinking of collagen fibrils, Collagen biosynthesis and formation. Out of the 9 collagen types, collagen- 1A1, 1A2, 6A1, 6A3, 9A1, 11A1 and 11A2 were all characterized as structural types that were part of the matrix^9^.

In contrast, collagen 12A1 and 14A1 are not part of the major structure. Collagen type 12A1 is thought to play a role in regeneration of tail and spinal cord in Notophthalmus viridescens^31^, and is involved in the regulation and organization of collagen fibril bundles^32^. Collagen type 14A1 has a regulatory role in early stage fibrillogenesis^33^, and plays a key role in regeneration in other tissues^34^. In addition, both Collagen 12a1 and 14a1, are involved in hydration and thickness, at least in other tissues^35^. At last, Collagen 15A1 has been characterized as a basement membrane protein that functions as a chondroitin sulfate proteoglycan^36^. This type of protein has multiple beneficial functions as described in the paragraphs on proteoglycans. Nevertheless, the evidence on all three collagen types is limited and more research on the functions of collagen 12A1, 14A1 and 15A1 in intervertebral discs is needed.

### Growth pathways

No proteoglycans were involved in any of the growth pathways. Out of the 5 selected glycoproteins, Thrombospondin 1, 2, 3, and 4 and Tenascin C were all involved in the ‘PI3K-Akt signaling pathway’. This pathway is involved in protein synthesis, inhibition of apoptosis and cell proliferation^37^, these three have shown to play major roles in disc degeneration^8^. Thrombospondins have been identified as a downstream effector protein in this pathway that regulates angiogenesis^38,39^. Interestingly, recent findings have illustrated that maintaining an anaerobe environment is crucial for the functioning of NP cells^40^, thereby highlighting the importance of regulating angiogenesis. In addition, both Tenascin C and thrombospondins activate TGF-β, which induces the PI3K-Akt signaling^39,41^, and thus makes their specific roles in the nucleus pulposus worth exploring.

Moreover, both tenascin C and Matrilin-3 were involved in the regulation of (IGF). This growth factor has shown to stimulate IVD cell proliferation and ECM synthesis^42^. Its expression rate is carefully regulated by Matrilin-3, thereby preventing overexpression^43^. At last, all collagen types characterized in the growth pathways were identified as structural collagens, and were targets in the pathways instead of initiators or regulators.

### Biological processes

With ten proteins involved, the most significantly upregulated biological process was ‘tissue development’. This process featured some proteins, which tissue development properties have been discussed earlier under the ECM assembly pathways: Collagen 1A1, 6A1, 11A1, 11A2 and 12A1. The remaining five proteins were involved in the development of a variety of tissues, some more relevant than others. Firstly, Tenascin C was involved in wound healing, during which it stimulates fibroblasts to increase the ECM protein synthesis^44^, thereby making it an attractive target for regenerative treatment. Interestingly, Thrombospondin 3 has proven to be important for osteogenesis, but its development functions in the NP remain unknown^45^. Moreover, TGFBI is involved in lumen formation during angiogenesis^46^. Because the nucleus pulposus homeostasis is maintained best in an anaerobic environment^40^, TGFBI might not be a preferential choice for regenerative therapies. The fourth protein involved was CTHRC1, which positively regulates bone formation^47^. Nonetheless, its overexpression is also associated with increased angiogenesis^48^, and a decrease in collagen type 1^49^, thereby questioning its practicability. The last protein engaged in tissue development was periostin, which is a protein participating in osteogenesis and post-injury tissue regeneration processes such as fibrosis and wound healing^50^. During these processes, periostin expression leads to degradation of ECM through upregulation of matrix metalloproteases^50^, which is reflected by an upregulation of periostin in degenerated discs^51^. Taken together, periostin seems very unsuitable for a regenerative therapy, and future treatments should focus on inhibiting this protein instead.

The second biological process identified was ‘cellular response to growth factor stimulus’, which included 5 proteins. As with the pathways, collagen 1A1 and 1A2 were substrates in this process^52^. Of the three remaining proteins, both fibromodulin and thrombospondin reflected their role in this process through binding to transforming growth factor-beta (TGF-β)^53^, thereby modulating its signal transduction. The third protein was periostin, which was regarded as unsuitable for regenerative therapies as mentioned above, it will not be discussed further.

The last upregulated biological process in fetal specific proteins was ‘regeneration’, which encountered 3 proteins. Out of which periostin’s and tenascin’s effects in wound healing have both been discussed above. At last, matrilin-2 it is known to promote muscle, neuron, liver and skeletal regeneration, which is mediated through TGF-β, bone morphogenetic protein 7 and Smad signaling^54^. Matrilins are generally multidomain extracellular adaptor proteins widely involved in the formation of both collagen-dependent & collagen-independent filamentous network^55^. Although functions of matrillin types are still unknown, matrillin-2 is the largest because of its two VWA domains that are connected by ten epidermal growth factor-like(EGF) modules^56^. They play a huge role in remodeling of tissue architecture, and they are widely expressed during differentiation and regeneration process in myogenic, neural and other tissues. It also plays a major role in tumorigenesis^54^.

Matrillin-2 can be detected in almost every other tissue type; they get secreted and deposited. They can be seen in loose and dense connective tissue, skeletal and smooth muscle, skin, subepithelial basement membrane, uterus, peripheral and central nervous system^55^. They function as interacting with other ECM molecules through their VWA domains to assemble into a filamentous network connected to collagen fibrils and proteoglycans^56^.

Study by Zhang et al., 2014 proposes matrillin-2 as a potent biomarker of Osteoarthritis in Articular Cartilage during its early stage^57^. For investigating the distribution patterns of matrillin-2, scientists have raised polyclonal antibodies specific to matrillin-2. With these antibodies they were able to detect two isoforms that are very specific to matrillin-2. Through immunohistochemistry, they were able to detect wide expression of matrillin-2 in loose & dense connective tissue, some specific epithelial cells and much inherently in some cartilage and bone^57^. By using a developmental animal model of Duncan-Hartley guinea pigs, the team also found increased expression of matrillin-2 in a damaged articular cartilage^57^. Role of matrilin-2 as an important ECM protein has been established in mice for proper and timely myogenic differentiation and skeletal muscle regeneration^58^. Unfortunately, its regenerative potential in disc disease is still unknown, as is the case with most discussed proteins. Nevertheless, Matrilin-2 seems to be a regenerative therapy candidate of high potential.

To summarize the relevance of the biological processes in addition to the pathways: The regenerative potential of tenascin C and fibromodulin was added upon with ECM assembly and interacting with TGF-β respectively. Most importantly, matrilin-2 clearly stood out due to its involvement in regeneration of many other tissues.

### Limitations

This study has several strongpoints: It was the first to compare the proteome of the human fetal and adult core matrisome. In addition, this study combined a qualitative, quantitative, statistical and pathway analysis in order to provide an overview of the findings and to put them in perspective in terms of future clinical applications. This study also has some limitations, even though multiple statistical tests were performed, no correction for multiple testing was implemented. This decision was made due to the explorative nature of the study and the low number of samples available. Nevertheless, some of the significant differences should therefore be interpreted with caution. Furthermore, the detection threshold for proteins identification of this study was limited by the sensitivity of mass spectrometer (q-value < 0.01). This limited sensitivity means that potentially interesting proteins with lower expression values may have been overlooked, and that proteins which are identified as unique in fetus could also be expressed in adult NP’s in very low quantities. This was illustrated by the validation through IHC, which is considered as a highly sensitive technique: Adult NP 2 showed a moderately positive staining for COL14a1 whereas none of the proteomic samples expressed COL14a1. Nevertheless, as very large differences in protein expression are of similar interest as uniquely expressed proteins, this limitation of proteomics should not be a major concern.

In short, this study provided an overview of the human fetal and healthy adult proteome and provided a list of fetal proteins of interest with potential for regenerative therapies for disc degeneration. In order to assess whether these potential candidates indeed have regenerative potential , future studies should focus on testing their regenerative capacities in mechanical experiments, in which an abundance of these proteins is added to adult discs to see if they can prevent degeneration, or added to degenerated discs to identify if they can reverse degeneration.

## Supplementary information

Supplementary Legends.

Supplementary Figure S1.

Supplementary Figure S2.
