# Supplementary material for: Part 1: profiling extra cellular matrix core proteome of human fetal nucleus pulposus in search for regenerative targets
Source: Sci Rep. 2020 Sep 24;10:15684. doi: 10.1038/s41598-020-72859-x (PMC7519061; doi:10.1038/s41598-020-72859-x)
Supplement: Supplementary file 1 — Supplementary Legends. [file 41598_2020_72859_MOESM1_ESM.docx]

**Part 1: Profiling extra cellular matrix core proteome of human fetal nucleus pulposus in search for regenerative targets.**

Shanmuganathan Rajasekaran Prof. MD.^1^, Chitra Thangavel PhD.^2^, Niek Djuric^2,3^, Muthurajan Raveendran Prof.^4^, Dilip Chand Raja Soundararajan ^1^, Sharon Miracle Nayagam^2^, Monica Steffi Matchado^2^, K S Sri Vijay Anand^1^

1. Department of Spine Surgery, Ganga Hospital, 313, Mettuppalayam Road, Coimbatore, 641043, India.

2. Ganga Research Centre, No 91, Mettuppalayam Road, Coimbatore, 641030, India.

3. Department of Neurosurgery, Leiden University Medical Center, Leiden

4 Department of Plant Biotechnology, Tamil Nadu Agricultural University, Coimbatore, 641003, India.

**Corresponding author:**

Shanmuganathan Rajasekaran, Department of Spine Surgery, Ganga Hospital, 313, Mettuppalayam Road, Coimbatore, 641043, India. 91-422-2485000, rajasekaran.orth@gmail.com

**Supplementary appendix**

Table S1

**Pfirrmann grade**Table S1 lists the degeneration scores (Pfirrmann grade) for all proteomic samples.

| **Sample number** | **Fetus** | **Healthy adult** | **Degenerated** |
| --- | --- | --- | --- |
| 1 | 1 | 3 | 4 |
| 2 | 1 | 2 | 5 |
| 3 | 1 | 2 | 5 |
| 4 | 1 | 2 | 4 |
| 5 | 1 | 2 | 4 |
| 6 | 1 | 2 | 5 |
| 7 | 1 | 2 | 4 |
| 8 | 1 |  | 4 |
| 9 | 1 |  | 4 |
| 10 |  |  | 5 |
| 11 |  |  | 5 |

Table S2
**Protein list and expression frequency**

Table S2 lists all core matrisomal proteins found in fetus and/or healthy adult NP’s. The number in indicates the number of samples in that group that expressed the respective proteins. The total number of samples was 9 for fetus and 7 for healthy adults. Missing values were excluded from the analysis.

| Protein name | Gene symbol | Cluster | Fetus | Healthy adult |
| --- | --- | --- | --- | --- |
| Aggreccan | ACAN | Proteoglycan | 9 | 7 |
| Asporin | ASPN | Proteoglycan | 1 | 1 |
| Biglycan | BGN | Proteoglycan | 9 | 7 |
| Chondroadherin | CHAD | Proteoglycan | 8 | 7 |
| Chondroitin sulfate proteoglycan 4 | CSPG4 | Proteoglycan | 3 | 0 |
| Decorin | DCN | Proteoglycan | 9 | 6 |
| Epiphycan | EPYC | Proteoglycan | 8 | 0 |
| Fibromodulin | FMOD | Proteoglycan | 8 | 7 |
| Hyaluronan and proteoglycan link protein 1 | HAPLN1 | Proteoglycan | 9 | 7 |
| Heparan sulfate proteoglycan | HSPG2 | Proteoglycan | 9 | 2 |
| Lumican | LUM | Proteoglycan | 8 | 7 |
| Osteoglycin | OGN | Proteoglycan | 8 | 7 |
| Osteomodulin | OMD | Proteoglycan | 1 | 2 |
| Proline and arginine rich end leucine rich repeat protein | PRELP | Proteoglycan | 9 | 7 |
| Proteoglycan 4 | PRG4 | Proteoglycan | 2 | 7 |
| Versican | VCAN | Proteoglycan | 7 | 7 |
| ABI family, member 3 (NESH) binding protein | ABI3BP | Glycoprotein | 8 | 7 |
| AE binding protein 1 | AEBP1 | Glycoprotein | 3 | 1 |
| Cartilage intermediate layer protein 1 | CILP | Glycoprotein | 1 | 7 |
| Cartilage intermediate layer protein 2 | CILP2 | Glycoprotein | 7 | 7 |
| Cartilage oligomeric matrix protein | COMP | Glycoprotein | 9 | 7 |
| Collagen triple helix repeat containing 1 | CTHRC1 | Glycoprotein | 7 | 0 |
| Dermatopontin | DPT | Glycoprotein | 1 | 5 |
| EGF-like repeats and discoidin I-like domains 3 | EDIL3 | Glycoprotein | 4 | 1 |
| Elastin microfibril interfacer 1 | EMILIN1 | Glycoprotein | 2 | 0 |
| Fibulins | FBLN1 | Glycoprotein | 4 | 0 |
| Fibrinogen alpha chain | FGA | Glycoprotein | 0 | 1 |
| Fibrinogen beta chain | FGB | Glycoprotein | 0 | 1 |
| Fibrinogen gamma chain | FGG | Glycoprotein | 0 | 1 |
| Fibronectin | FN1 | Glycoprotein | 7 | 7 |
| Fibronectin type III domain containing 1 | FNDC1 | Glycoprotein | 6 | 0 |
| Gliomedin | GLDN | Glycoprotein | 3 | 0 |
| Matrilin-2 | MATN2 | Glycoprotein | 6 | 0 |
| Matrilin-3 | MATN3 | Glycoprotein | 9 | 0 |
| Matrilin-4 | MATN4 | Glycoprotein | 4 | 0 |
| Milk fat globule-EGF factor 8 protein | MFGE8 | Glycoprotein | 6 | 5 |
| Nidogen 1 | NID1 | Glycoprotein | 1 | 0 |
| Nidogen 2 | NID2 | Glycoprotein | 8 | 0 |
| Procollagen C-endopeptidase enhancer | PCOLCE | Glycoprotein | 7 | 3 |
| Procollagen C-endopeptidase enhancer 2 | PCOLCE2 | Glycoprotein | 0 | 2 |
| Periostin, osteoblast specific factor | POSTN | Glycoprotein | 6 | 0 |
| Sushi-repeat-containing protein, X-linked | SRPX | Glycoprotein | 4 | 0 |
| Sushi-repeat-containing protein, X-linked 2 | SRPX2 | Glycoprotein | 7 | 2 |
| Transforming growth factor-beta-induced protein ig-h3 | TGFBI | Glycoprotein | 8 | 5 |
| Thrombospondin 1 | THBS1 | Glycoprotein | 9 | 1 |
| Thrombospondin 3 | THBS3 | Glycoprotein | 6 | 0 |
| Thrombospondin 4 | THBS4 | Glycoprotein | 6 | 0 |
| Tenascins | TNC | Glycoprotein | 8 | 0 |
| Tumor necrosis factor, alpha-induced protein 6 | TNFAIP6 | Glycoprotein | 0 | 2 |
| Vitronectin | VTN | Glycoprotein | 0 | 1 |
| Collagen, type I, alpha 1 | COL1A1 | Collagen | 3 | 0 |
| Collagen, type I, alpha 2 | COL1A2 | Collagen | 4 | 0 |
| Collagen, type II, alpha 1 | COL2A1 | Collagen | 8 | 7 |
| Collagen, type VI, alpha 1 | COL6A1 | Collagen | 9 | 6 |
| Collagen, type VI, alpha 2 | COL6A2 | Collagen | 9 | 3 |
| Collagen, type VI, alpha 3 | COL6A3 | Collagen | 9 | 7 |
| Collagen, type IX, alpha 1 | COL9A1 | Collagen | 8 | 0 |
| Collagen, type IX, alpha 2 | COL9A2 | Collagen | 1 | 0 |
| Collagen, type X, alpha 1 | COL10A1 | Collagen | 1 | 0 |
| Collagen, type XI, alpha 1 | COL11A1 | Collagen | 7 | 0 |
| Collagen, type XI, alpha 2 | COL11A2 | Collagen | 9 | 7 |
| Collagen, type XII, alpha 1 | COL12A1 | Collagen | 9 | 0 |
| Collagen, type XIV, alpha 1 | COL14A1 | Collagen | 9 | 0 |
| Collagen, type XV, alpha 1 | COL15A1 | Collagen | 4 | 1 |

**Supplementary figure S1**

**Cytoscape string analysis of biglycans outgoing signals**Grey strings show interactions between proteins, all gene symbols in blue boxes receive incoming signals from biglycan.

**Supplementary figure S2**

**Cytoscape string analysis of biglycans incoming signals**

Grey strings show interactions between proteins, all gene symbols in blue boxes send outgoing signals to biglycan.
