# Supplementary figures and images for: Part 1: profiling extra cellular matrix core proteome of human fetal nucleus pulposus in search for regenerative targets
Source: Sci Rep. 2020 Sep 24;10:15684. doi: 10.1038/s41598-020-72859-x (PMC7519061; doi:10.1038/s41598-020-72859-x)

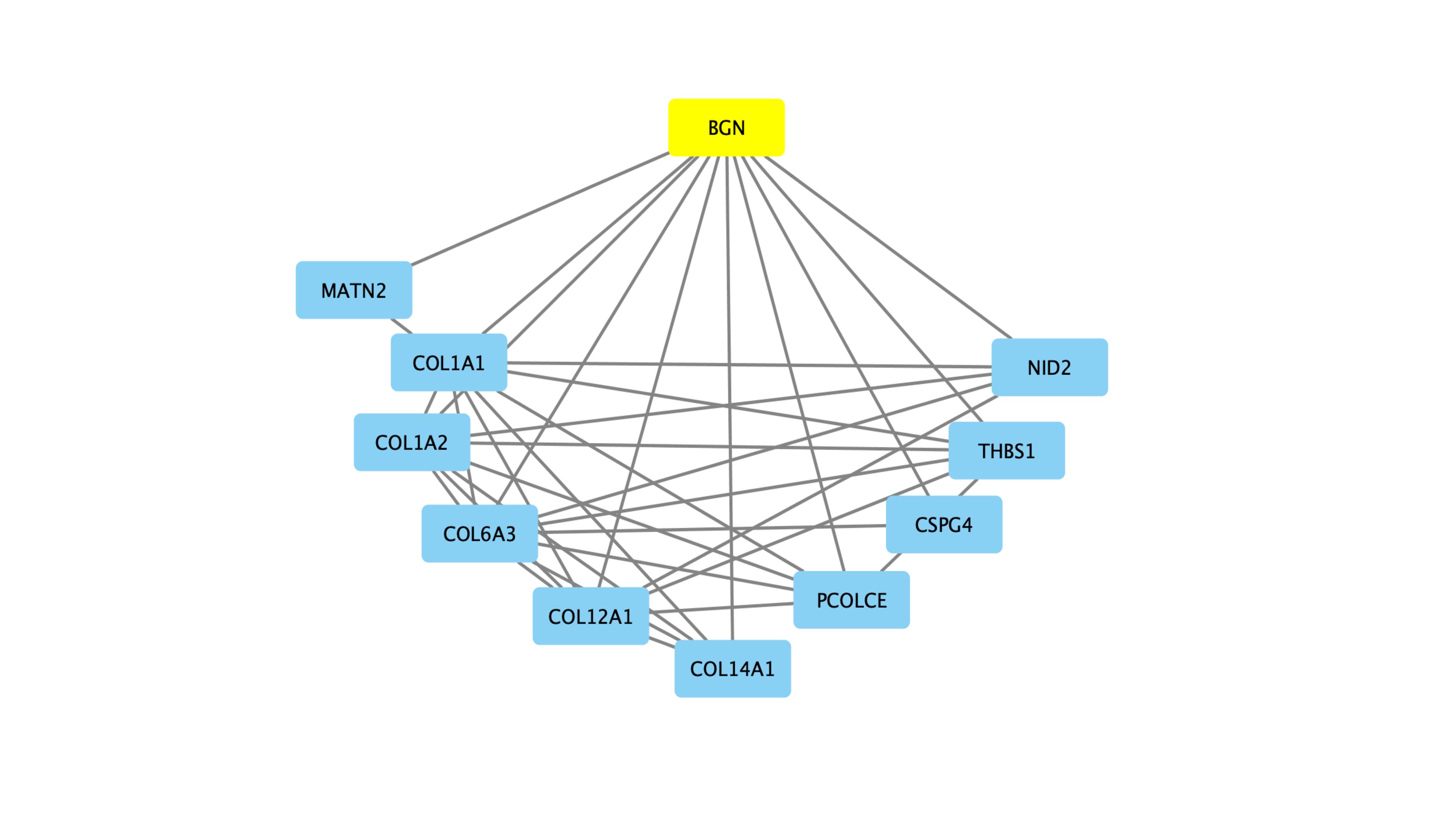

Supplement: Supplementary file 2 — Supplementary Figure S1. [file 41598_2020_72859_MOESM2_ESM.jpg]

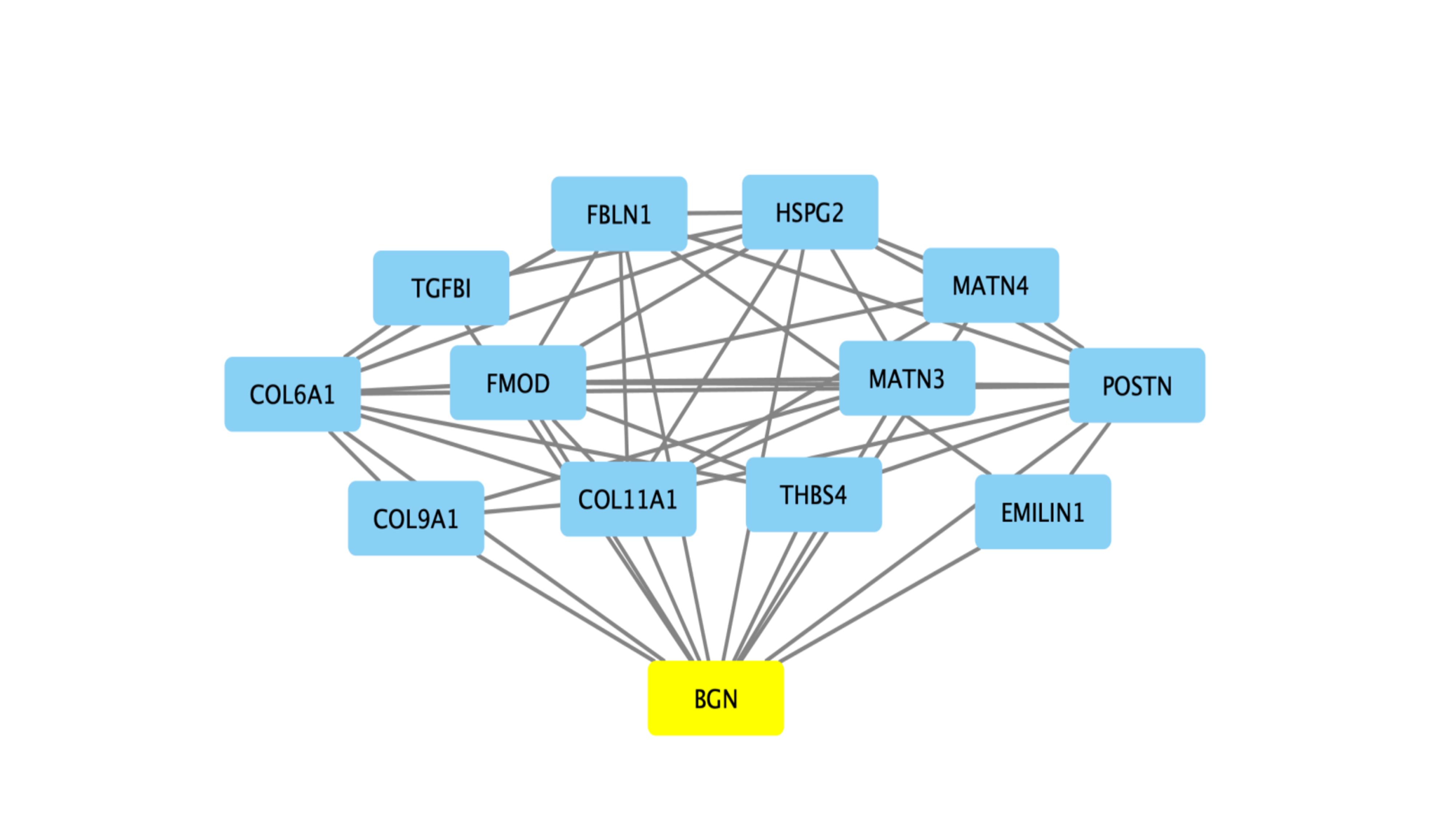

Supplement: Supplementary file 3 — Supplementary Figure S2. [file 41598_2020_72859_MOESM3_ESM.jpg]
